# A latitudinal gradient in climate effects on seabird demography: results from interspecific analyses

**DOI:** 10.1111/j.1365-2486.2007.01533.x

**Published:** 2008-04

**Authors:** HANNO SANDVIK, TIM COULSON, BERNT-ERIK SÆTHER

**Affiliations:** * Division of Biology, Imperial College LondonSilwood Park, Ascot, Berkshire SL5 7PY, UK; † Centre for Conservation Biology, Department of Biology, Norwegian University of Science and Technology (NTNU)7491 Trondheim, Norway

**Keywords:** breeding success, demography, interspecific analysis, latitude, North Atlantic Oscillation, sea surface temperature

## Abstract

For an understanding of the effect of climate change on animal population dynamics, it is crucial to be able to identify which climatologic parameters affect which demographic rate, and what the underlying mechanistic links are. An important reason for why the interactions between demography and climate still are poorly understood is that the effects of climate vary both geographically and taxonomically. Here, we analyse interspecifically how different climate variables affect the breeding success of North Atlantic seabird species along latitudinal and longitudinal gradients. By approaching the problem comparatively, we are able to generalize across populations and species. We find a strong interactive effect of climate and latitude on breeding success. Of the climatic variables considered, local sea surface temperatures during the breeding season tend to be more relevant than the North Atlantic Oscillation (NAO). However, the effect of NAO on breeding success shows a clear geographic pattern, changing in sign from positive in the south to negative in the north. If this interaction is taken account of, the model explains more variation than any model with sea surface temperature. This superiority of the NAO index is due to its ability to capture effects of more than one season in a single parameter. Mechanistically, however, several lines of evidence suggest that sea surface temperature is the biologically most relevant explanatory variable.

## Introduction

Climate can have profound effects on the demography and population dynamics of marine top predators. Whereas mass mortality and breeding failure of South Pacific seabirds in El Niño years is a recurrent and comparatively well understood phenomenon (Barber & Chavez, 1983; Schreiber & Schreiber, 1984; Duffy, 1990; Schreiber, 2002;), the interaction between climate and top predators in oceanic regions that are not governed by the El Niño-Southern Oscillation, are far more subtle.

In the North Atlantic Ocean it has been shown that many life history and other characteristics of seabirds correlate well with the North Atlantic Oscillation (NAO) index (Reid *et al.*, 1999; Durant *et al.*, 2004; Grosbois & Thompson, 2005; Sandvik *et al.*, 2005; Sandvik & Erikstad, 2008;). The NAO is defined as sea level pressure anomalies over the North Atlantic Ocean (Walker, 1924; Hurrell *et al.*, 2003;). As such, the NAO can hardly be said to be the direct cause of any biological phenomenon. The widespread statistical associations of biological parameters with the NAO can, thus, be viewed both as a strength and as a call for more research (Hallett *et al.*, 2004; Stenseth & Mysterud, 2005;): on the one hand, the NAO index is a useful proxy which seems to integrate information on a temporal and spatial scale that is biologically relevant for many species. On the other hand, it is often still an open question what this biologically relevant information is, because local weather repeatedly performs more poorly as an explanatory variable of ecological processes. In some well-studied terrestrial, freshwater and marine systems, it has been possible to reveal the underlying causal pathways and to disentangle the interactions of climate with geography, topography or food-web structure (e.g. Post & Stenseth, 1999; Hjermann *et al.*, 2004;). In other cases, one still knows little more than that NAO and biological characteristics of study species covary. However, knowledge of the underlying mechanisms is crucial for an understanding of the effects global warming will have on the population dynamics of biological species.

**Fig. 1:**
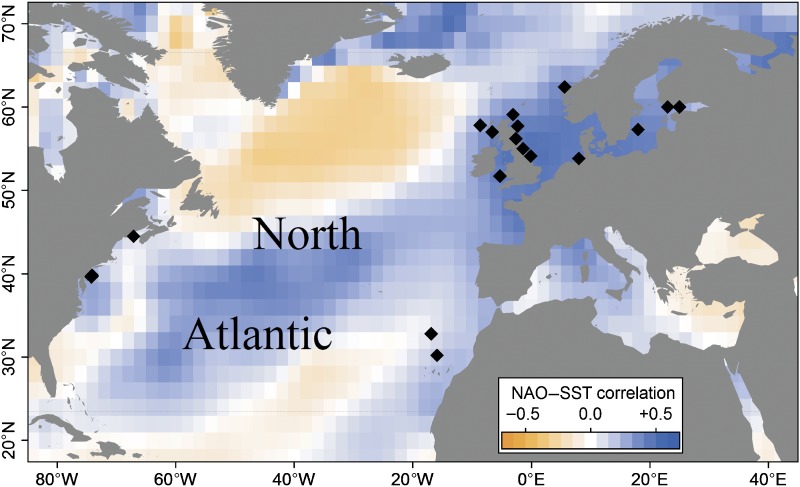
A map of the North Atlantic showing the locations of the study colonies (black diamonds). The colour patterns visualize the local correlation between NAO and the local SST eight months earlier.

In seabirds, the climatic parameter relevant for demography has been hypothesized, and in some study systems substantiated, to be sea surface temperature (SST; Peck *et al.*, 2004; Harris *et al.*, 2005; Sandvik *et al.*, 2005;), which is strongly correlated with NAO. However, both the exact magnitude and even the sign of the NAO–SST correlation vary geographically throughout the North Atlantic (Hurrell *et al.*, 2003; cf. Fig. 1). This poses a severe obstacle to understanding the relationships between climate and demography, because the climatic responsiveness of animal demographic parameters likewise can vary considerably across different geographical areas (Mysterud *et al.*, 2000; Sæther *et al.*, 2003, 2004; Harris *et al.*, 2005; Sandvik & Erikstad, 2008). The study of climatic responsiveness in seabird demography is complicated by two further factors. First, seabirds are pelagic organisms spreading over vast oceanic regions where they are extremely difficult to study. This causes difficulties in choosing the appropriate scale at which environmental covariates should be considered. Second, the main effect of SSTs has been shown to be indirect [i.e. to be mediated through the food chain via prey abundance and/or prey availability rather than being the result of weather conditions on seabirds themselves (Weimerskirch *et al.*, 2003; Abraham & Sydeman, 2004; Durant *et al.*, 2004; Sandvik *et al.*, 2005;)]. This may introduce time lags into the interaction between climate and demography that can be difficult to identify.

In this study, we investigate whether SST or NAO is better able to capture the effect of climate on seabird breeding success. We also search for geographical patterns in the covariation between seabird breeding success and NAO and/or SST. Those questions are necessary for a better causal and mechanistic understanding of the link between climate and demography. We approach these problems by analysing an interspecific dataset, utilizing available time series on temporal variation in breeding success in the North Atlantic. Addressing the issue comparatively, makes the study nomothetic rather than merely descriptive (i.e. it generalizes across species). The results demonstrate that the climatic response in seabird demography is indeed geographically patterned, among other things because it changes its sign with latitude.

## Materials and methods

We surveyed the literature for studies reporting breeding success of seabirds in the North Atlantic for at least four consecutive years. ‘Seabird’ was defined as any species belonging to the Alcinae (auks), Larinae (gulls), Procellariidae (petrels and fulmars), Sterninae (terns) or Sulidae (gannets). Breeding success was defined as the proportion of eggs laid that survived until fledging of the chicks. The estimates of breeding success were logit-transformed and standardized. The final data set contained 33 studies on 13 species, differing in length between 4 and 29 years (see Appendix, Table A1). The locations of the colonies are shown in Fig. 1.

Two sorts of analyses were performed. Initially data were analysed using linear mixed effects models. In these analyses, the transformed time series of breeding success were the dependent variables; NAO indices and SSTs at different time lags, latitude, longitude, body mass, clutch size, time series length and a binary variable ‘America vs. Europe’ were considered as fixed effects; whereas colony, cluster of colonies (cf. Table A1), species, higher taxon (as delineated in the first paragraph of ‘Materials and methods’), and/or year were treated as potential random effects. The main aim of these analyses was to identify the climatic time lags that were most relevant.

The interrelationships between breeding success, climate and geography at those time lags were then analysed in greater detail using derived variable analyses. This method entailed estimating for each breeding colony the slope of breeding success against environmental variables (NAO and SSTs) in univariate linear models. Because both the dependent and the explanatory variable were standardized, the slopes were equivalent to correlation coefficients (*r*). These slopes were then treated as dependent variables and, after *z*-transformation (Sokal & Rohlf, 1995, p. 757), analysed further using linear models, weighting each slope by the length of the underlying time series. The explanatory variables considered were latitude, longitude, body mass, clutch size, time series length, higher taxon and the local correlation between NAO and SST. The latter variable was defined as the product–moment correlation between winter (December–March) NAO and the local summer (April–July) SST around each breeding colony. The time interval chosen for estimating local NAO–SST correlation was the 30-year-period 1965–1994, because all studies overlapped with this time frame. The time lags considered for those correlations were −1 to +1 [i.e. correlations between NAO and SSTs of the preceding summer (SST leading 8 months), of the same summer (NAO leading by 4 months), and of the following summer (NAO leading by 16 months)].

Model simplification was guided by Akaike's Information Criterion corrected for small sample sizes (AIC_C_). As standard models make a number of implicit statistical assumptions, which may or may not be fulfilled (such as absence of spatial autocorrelation and of density dependence, homogeneous variances, etc.), we run several variations of the final set of preferred models in order to test their robustness. Those variations are listed in Appendix A (Table A2) and discussed in the text only to the degree they differ from the standard models.

The NAO was represented by the principal component-based extended winter (December–March) NAO index (Hurrell, 2005) lagged by zero to two years (i.e. 4, 16 and 28 months before the respective breeding season). Sea surface temperatures (ERSST, Smith & Reynolds, 2006) were averaged for areas of a 250 km radius around each breeding colony for the breeding season (April–July) and the preceding winter (December–March) lagged by 0–2 years. All time series were standardized for each colony. Mean body masses and clutch sizes of each species were taken from Schreiber & Burger (2002).

All analyses were performed in the R environment (R development core team, 2005). Estimates are given as mean±standard error.

## Results

### Linear mixed effects modelling

Linear mixed effects models with colony as random effect were used to analyse the factors affecting breeding success. Higher taxa, species, clusters of colonies, and year increased the models' AIC_C_, and could be dropped as random effects. Among the explanatory variables (fixed effects), longitude, continent (i.e. America vs. Europe), body mass, clutch size, time series length and year did not explain the variation in breeding success. The only time lags at which climatic variables were retained in any model, was at lag 1 for NAO and at time lag 0 for summer SST (Table 1). A model containing these two variables, although not significant, shows that the slopes of breeding success on SST are almost five times larger than the slopes of breeding success on NAO (Table 1). The sign of the effects indicates that breeding success increases with increasing water temperature during the current breading season, and decreases with increasing NAO index during the winter 1 year earlier. In contrast, the effects of winter SSTs are insignificant. Several models with only main effects are considerably better supported than the null model (negative ΔAIC_C_ in Table 1). However, in none of these are the effects statistically significant. If allowing for interactions, on the other hand, both the main climate affect and the interaction with latitude turn out to be significant – highly so in the case of NAO (Table 1). The models explain up to 4.9% of the variance in breeding success.

**Table 1:** Effects of different climatic variables on the breeding success of North Atlantic seabirds

Model/parameter	Estimate ± SE	*F/t*	*p*	df	*r*^2^	ΔAIC_C_
**SST**		**1.96**	**0.15**	**1, 289**	**0.01**	**−6.14**
SST_S, 0_	+0.10 ± 0.07	1.45	0.15	289		
**NAO**		**0.36**	**0.80**	**1, 289**	**0.00**	**−4.61**
NAO_W, 1_	**−**0.02 ± 0.07	−0.25	0.80	289		
**NAO+SST**		**1.22**	**0.30**	**2, 288**	**0.01**	**−6.40**
NAO_W, 1_	**−**0.02 ± 0.07	−0.30	0.76	288		
SST_S, 0_	+0.11 ± 0.07	1.51	0.13	288		
**SST including winter**		**0.97**	**0.38**	**2, 288**	**0.01**	**−2.20**
SST_S, 0_	+0.11 ± 0.07	1.62	0.106	288		
SST_W, 1_	+0.03 ± 0.06	0.54	0.59	288		
**NAO × latitude**		**5.44**	**0.0012**	**3, 287**	**0.05**	**−12.09**
NAO_W, 1_	+1.16 ± 0.33	3.53	0.00049	288		
Latitude	0.00 ± 0.00	0.00	1.0	32		
NAO_W, 1_× latitude	**−**0.02 ± 0.01	**−**3.68	0.00028	288		
**SST × latitude**		**2.65**	**0.049**	**3, 287**	**0.02**	**−6.57**
SST_S, 0_	+0.90 ± 0.38	2.36	0.019	288		
Latitude	0.00 ± 0.00	0.00	1.0	32		
SST_S, 0_× latitude	**−**0.02 ± 0.01	**−**2.12	0.035	288		

Statistics provided are for the linear mixed effects models as such (boldface: *F* statistic, significance level, degrees of freedom, variance explained, and Akaike's Information criterion corrected for small sample size) and for their fixed effects (parameter estimate, Student's *t*, significance level). Localities of the breeding populations were treated as random factors. The environmental variables considered are North Atlantic Oscillation (NAO) index and sea surface temperatures (SST); subscripts indicate the season (S=summer, W=winter) and the time lag (i.e. the number of years the climatic variables predate the relevant breeding seasons). ΔAIC_C_ values are expressed as the difference between the AIC_C_ of a model and the AIC_C_ of the constant model (i.e., without covariates), where low values indicate better models.

### Derived variables analyses

**Fig. 2:**
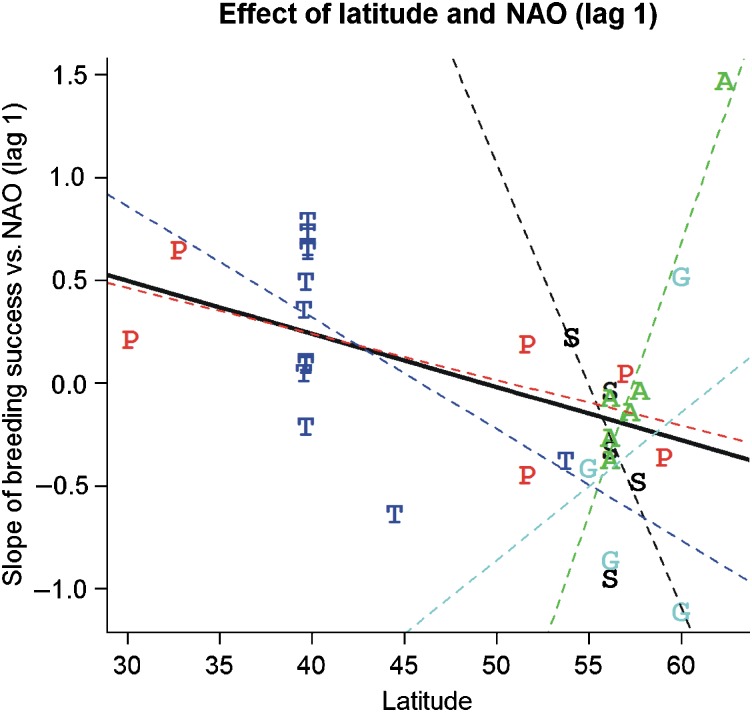
Effect of latitude and winter NAO on breeding success 16 months later in different seabird taxa. Overall, the slope of breeding success against NAO decreases towards the north. However, there are pronounced differences between taxa. The slopes (*y* axis) are equivalent to *z*-transformed correlation coefficients. Legend: bold solid line, all taxa; green A, auks; turquois G, gulls; red P, petrels (incl. fulmar); black S, sulids (gannet); dark blue T, terns.

The mean slopes of breeding success against winter NAO (lagged 1 year) and against summer SSTs (unlagged) are +0.041±0.111 and +0.044±0.110, respectively, when derived from the same bivariate model. The mean-squared slopes (which are equivalent to coefficients of determination, *r*^2^) are 0.38±0.14 and 0.38±0.09. If using univariate models (i.e. if not controlling the effect of the other explanatory variable), the mean (squared) slopes are 0.000±0.094 (0.20±0.04) against NAO and +0.110±0.083 (0.22±0.04) against SST. Linear models were used to analyse the variation in the latter slopes. Latitude alone explains 26% of this variation (Table 2, Fig. 2). The respective model is well supported. If an independent class variable that denotes higher taxon (with the levels Alcinae, Larinae, Procellariidae, Sterninae and Sulidae), and its interaction with latitude, is included in the analysis, the proportion of the variance explained is doubled (Fig. 2). However, whereas the taxon and interaction effects are marginally significant (anova, *F*_8, 23_=2.34, *P*=0.053), the high number of parameters leads to its rejection according to AIC_C_ (Table 2).

**Table 2:** Factors affecting the slopes of seabird breeding success against winter NAO 16 months earlier

Model/parameter	Estimate ± SE	*F/t*	*p*	df	*r*^2^	ΔAIC_C_
**Latitude**		**10.95**	**0.0024**	**1, 31**	**0.26**	**−7.56**
Intercept	+1.27 ± 0.40	3.17	0.0034			
Latitude	−0.03 ± 0.01	**−**3.31	0.0024			
**Latitude × taxon**		**3.72**	**0.0052**	**9, 23**	**0.59**	**+0.52**
Intercept	−15.13 ± 5.14	**−**2.95	0.0073			
Latitude	+0.26 ± 0.09	2.93	0.0075			
Larinae	+10.67 ± 6.45	1.65	0.11			
Procellariidae	+16.27 ± 5.16	3.15	0.0045			
Sterninae	+17.62 ± 5.22	3.37	0.0026			
Sulidae	+27.01 ± 8.70	3.11	0.0050			
Latitude × Larinae	−0.19 ± 0.11	**−**1.69	0.10			
Latitude × Procellariidae	−0.29 ± 0.09	**−**3.16	0.0044			
Latitude × Sterninae	−0.32 ± 0.09	**−**3.43	0.0023			
Latitude × Sulidae	−0.48 ± 0.15	**−**3.10	0.0051			
**Local correlation with SST**		**15.28**	**0.00047**	**1, 31**	**0.33**	**−10.80**
Intercept	+0.76 ± 0.21	3.54	0.0013			
SST correlation	−2.13 ± 0.55	**−**3.91	0.00047			

Statistics provided are for the linear models as such (boldface) and for the model parameters. See Table 1 for further explanations.

**Fig. 3:**
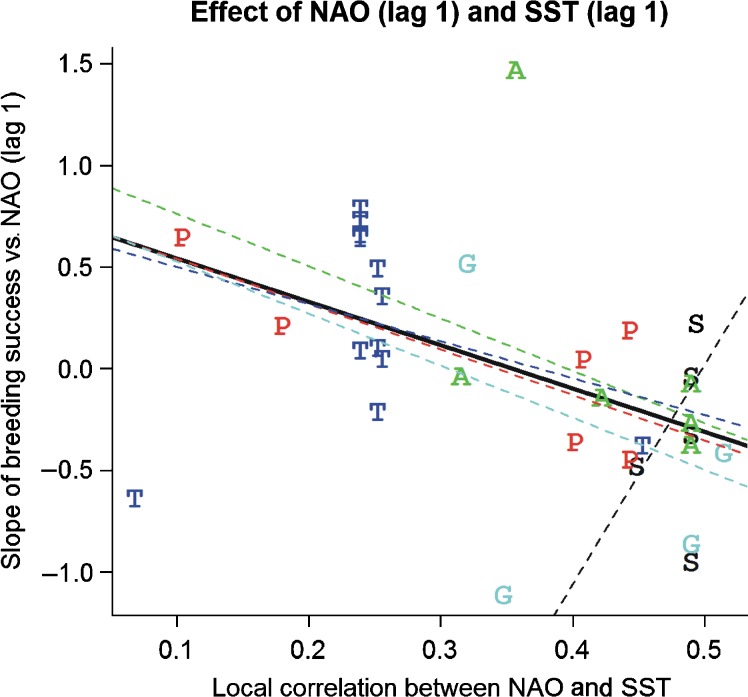
The combined effect of NAO and SST on breeding success in different seabird taxa. The stronger the local correlation between NAO and SST, the more negative is the slope of the regression of breeding success on NAO. Taxonomic differences are weak to absent. The slopes (*y* axis) are equivalent to *z*-transformed correlation coefficients. Legend: bold solid line, all taxa; green A, auks; turquois G, gulls; red P, petrels (incl. fulmar); black S, sulids (gannet); dark blue T, terns.

When introducing the local correlation between winter NAO and the preceding summer SSTs (i.e. lag+1 year) as an additional explanatory variable, it turned out that a model with only that variable achieves the lowest (i.e. best) AIC_C_ (Table 2, Fig. 3). All local NAO–SST correlations are positive (Figs 1 and 2). None of the other explanatory variables considered (longitude, body mass, clutch size, time series length) are relevant (all *P*>0.50). Neither do the other time lags of the local correlation improve the model (lag 0, ΔAIC_C_=−6.99; lag−1 year, ΔAIC_C_=+1.09). The difference between taxa is negligible (ΔAIC_C_=+3.89; anova, *F*_8, 23_=0.32, *P*=0.95; cf. Fig. 3).

The results are very robust to changes in the analysis methods (Appendix A, Table A2). Under all modifications, the model with local NAO–SST correlation achieves the lowest AIC_C_. The latter models (all *F*>5.5, all *P*<0.03), and the ones with latitude as explanatory variable (all *F*>3.5, all *P*<0.08), are at least marginally significantly supported (Table A1). The interactive model (latitude × taxon) is significant in half of the cases (all *F*>1.7, all *P*<0.18), even though its support as measured by AIC_C_ or likelihood ratio tests is much poorer than for the other two models.

The variance in slopes of breeding success on SST of the previous summer is only poorly explained by the variables considered (Table 3). The best model according to the AIC_C_ contained latitude, the local correlation between SST and NAO, and the interaction between these two variables (Table 3). However, all models contain nonsignificant factors, and none of them would have been preferred over the constant model (with no covariates) according to an anova.

**Table 3:** Factors affecting the slopes of seabird breeding success against unlagged summer SST

Model/parameter	Estimate ± SE	*F/t*	*p*	df	*r*^2^	ΔAIC_C_
**Latitude**		**3.08**	**0.089**	**1, 31**	**0.09**	**−0.70**
Intercept	+1.04 ± 0.53	1.96	0.059			
Latitude	**−**0.02 ± 0.01	**−**1.76	0.089			
**Local correlation with NAO**		**0.07**	**0.79**	**1, 31**	**0.00**	**+2.35**
Intercept	+0.20 ± 0.31	0.65	0.52			
NAO correlation	**−**0.21 ± 0.79	**−**0.26	0.79			
**Longitude**		**3.64**	**0.066**	**1, 31**	**0.11**	**−1.24**
Intercept	**−**0.02 ± 0.11	**−**0.21	0.83			
Longitude	**−**0.01 ± 0.00	**−**1.91	0.066			
**Latitude and local correlation with NAO (additive)**		**3.65**	**0.038**	**2, 30**	**0.20**	**−2.16**
Intercept	1.48 ± 0.55	2.67	0.012			
Latitude	**−**0.04 ± 0.02	**−**2.69	0.012			
NAO correlation	2.40 ± 1.21	1.98	0.057			
**Latitude and local correlation with NAO (multiplicative)**		**3.86**	**0.019**	**3, 29**	**0.29**	**−3.26**
Intercept	**−**1.76 ± 1.78	**−**0.99	0.33			
Latitude	0.02 ± 0.04	0.61	0.54			
NAO correlation	16.07 ± 7.27	2.21	0.035			
Latitude × NAO correlation	**−**0.27 ± 0.14	**−**1.91	0.067			

Statistics provided are for the linear models as such (boldface) and for the model parameters. See Table 1 for further explanations.

## Discussion

In an interspecific analysis of 33 time series from 13 species of North Atlantic seabirds, we demonstrate that the effect of NAO on breeding success shows a strong geographic pattern. When only considering main effects, SST accounts for a considerably larger amount of variance in breeding success among colonies than NAO (Table 1). However, a far better model can be obtained by including latitude and the interaction between latitude and NAO. This shows that the effect of NAO on breeding success becomes increasingly negative towards the north (Fig. 2), a conclusion that mirrors earlier findings in hole-nesting birds (Sæther *et al.*, 2003). If geography is ignored, the positive correlations between breeding success and NAO in the southern colonies on the one hand, and the negative correlations in the northern colonies on the other hand, effectively cancel each other out. This can be seen by comparing the mean slopes (0.00±0.09) with the mean squared slopes (+0.20±0.04, equivalent to an *r*^2^). Only in the interaction with latitude, the effect of NAO becomes apparent (Fig. 2). Such a latitudinal pattern is not found for the effect of SST on breeding success (Table 1).

Analyses of the slopes of breeding success against the two major explanatory variables, NAO and SST, confirmed those findings. 26% of the variation in the slopes of breeding success on NAO could be accounted for by latitude alone (Table 2). The proportion was doubled when differences between five higher taxa of seabirds (auks, gulls, terns, petrels, and gannets) were included into the model (Fig. 2). The latter model was poorly supported, however. This is probably because several of the taxa are represented from quite narrow latitudinal ranges only (Fig. 2), which increases the uncertainty of their slopes. The effect of latitude remained at least marginally significant in all – and highly significant in most – of the modified models (Table A2). The variances in slopes explained by latitude varied between 16% and 39%, depending on the statistical assumptions made.

Although SSTs were at least as important for breeding success as NAO, the variance in slopes of breeding success against SST were less readily accounted for by the covariates considered (Table 3). In other words, the relationship between SST and breeding success does not vary much across latitudes or taxa. This can, of course, have many reasons. However, it rather strengthens than weakens the interpretation that SST is an important climatic variable affecting seabird breeding success (e.g. Murphy & Schauer, 1994; Guinet *et al.*, 1998; Gjerdrum *et al.*, 2003; Inchausti *et al.*, 2003; Frederiksen *et al.*, 2004; but see Jenouvrier *et al.*, 2003). Assuming that SST indeed is the single most important nonbiological variable, one cannot expect the remaining variables to explain the residual variation after the effect of SST is taken into account.

The signs of the correlations between SST (unlagged) and breeding success are positive. This would be compatible with a direct effect of weather on breeding performance (i.e. an increased mortality of chicks in colder-than-average breeding seasons). Alternatively, the effect of SST is mediated through the food chain. The availability of many marine prey species of seabirds is affected by SST, even though the signs of those correlations vary with species and region (Alheit & Hagen, 1997; Ottersen & Loeng, 2000; Genner *et al.*, 2004; Hjermann *et al.*, 2004; Cook & Heath, 2005; Rose, 2005;). Finally, it may be the case that both summer SST and the performance of adult breeders are correlated with a third factor, such as weather conditions during the winter before the respective breeding attempt, operating through an effect on body condition (e.g. Durant *et al.*, 2006). However, this interpretation is unlikely because of the absence of any clear effect of winter SST on breeding success (Table 1).

A number of earlier studies has found negative correlations between SST and breeding success. These results are not in direct conflict with our positive relationship, however, because the other studies either stem from the Pacific (Kitaysky & Golubova, 2000; Gaston & Smith, 2001; Gjerdrum *et al.*, 2003; Smithers *et al.*, 2003;), where warm water events co-occur with collapses in the upwelling systems; from the Southern Ocean (Inchausti *et al.*, 2003; Jenouvrier *et al.*, 2003;), where many seabirds feed at the ice edge; or considered SST at higher time lags (Frederiksen *et al.*, 2004). On the other hand, Durant *et al.* (2003, 2006) reported a positive relationship between SST and breeding success of Atlantic puffins (*Fratercula arctica*) in the Norwegian Sea.

According to the above interpretation, the significant effect of NAO on the breeding success of the following year may be predominantly due to the correlation between NAO and SST. Other meteorological parameters that covary with the NAO, such as precipitation and wind speed, are of less importance for marine organisms. This would make sense of the finding that models with NAO only become significant when latitude is included as a covariate. The reason is that the covariation between NAO and SST is geographically variable (‘tripole’ pattern; cf. Hurrell *et al.*, 2003; Hurrell & Dickson, 2004; Cohen & Barlow, 2005;Fig. 1), so that the same NAO index value has different oceanographic and, thus, biological meanings in different parts of the North Atlantic Ocean. If this view is correct, however, it is not latitude *per se* that interacts with NAO to influence breeding success, but the correlation between NAO and SST. To test this hypothesis, we also carried out analyses with local NAO–SST correlations as covariates. Those were the best supported models in all cases and under all statistical assumptions (Tables 2 and A2). Figure 3 illustrates this relationship: with an increasing local correlation between NAO and SST, the slope of breeding success on NAO decreases. This decline is surprisingly coherent across the different taxa of seabirds.

Overall, however, the model incorporating both NAO and latitude performed better than the SST models (Table 1), suggesting that most, but not all, relevant climatic information is contained in the SST variable (cf. Hallett *et al.*, 2004; Stenseth & Mysterud, 2005;). This is also visible from the fact that the intercept in Fig. 3 is significantly larger than zero: in the absence of any strong correlation between NAO and SST, NAO can still explain noteworthy amounts of variation in breeding success. Those are, by inference, unrelated to SSTs, at least at the time lag considered. What the causal links are in these cases is still an open question. On the other hand, when NAO and SST are correlated (right-hand side of Fig. 3), negative NAO values 1 year earlier (and, thus, low SSTs 2 years earlier) coincide with high breeding success. This, in turn, illustrates that one obvious advantage of the NAO index, methodologically speaking, is that it captures information from several seasons. Mechanistically speaking, however, even this larger amount of biological variance explained seems to be due to a large degree to SSTs, only at different time lags (positive at lag 0, negative at lags 1 and especially 2).

The time lags at which effects of NAO and SST were considered, were between zero and two years. The strongest effects were found at a time lag of zero and 1 year (i.e. breeding success was most strongly influenced by SSTs during the breeding season, and by the winter NAO 16 months before the breeding season). The correlation between December–March NAO and April–July SST was estimated for time lags between −1 and +1 years (or, more specifically, for time lags of −16, −4 and +8 months). The correlation that proved most relevant was the one in which the SST preceded NAO by 8 months (cf. Fig. 1). This is in accordance with oceanographic and climatological findings that the long-term atmospheric patterns constituting the NAO are forced by SSTs rather than the other way around (Wang *et al.*, 2004). The presence of these time lags indicates that the effect of climate cannot be restricted to a direct influence of weather on seabird breeding success. Instead, an influence on the local food source around the breeding colony is suggested. The negative sign of the lagged correlations are compatible with many findings of poorer prey abundance or prey recruitment in warmer-water years (Alheit & Hagen, 1997; Frederiksen *et al.*, 2004; Hjermann *et al.*, 2004; Cook & Heath, 2005;). This effect might carry over to the following breeding season(s) both because seabirds often prey upon earlier fish cohorts (e.g. Hjermann *et al.*, 2004) and by affecting the body condition of breeders (e.g. Frederiksen *et al.*, 2004). In order to get a better understanding of the precise size of the time lags, it might be an interesting exercise to incorporate the seabirds' major prey species into future comparative analyses (cf. Kitaysky & Golubova, 2000).

The effect of climatic variables on breeding success documented in this study is compatible with the ‘tap’ hypothesis (Sæther *et al.*, 2004), which poses that climate influences population dynamics by affecting recruitment (Crespin *et al.*, 2006) rather than survival (‘tub’ hypothesis). As long-lived species with a comparatively low reproductive output per breeding attempt, seabirds have been hypothesized to be more strongly affected by climatic effects on survival (Lebreton & Clobert, 1991; Wooller *et al.*, 1992;). Previous findings are in accordance with this assumption (Grosbois & Thompson, 2005; Harris *et al.*, 2005; Sandvik *et al.*, 2005; cf. Sandvik & Erikstad, 2008). As the tap and tub hypothesis are not mutually exclusive, the current findings do of course not contradict that evidence, and indicate that population dynamics even of long-lived birds may be affected by climate both through a tap and a tub effect.

To conclude, we have shown the intercolony variation of breeding success of North Atlantic seabirds to be related to both NAO and SST. The main abiotic determinant is hypothesized to be SST, because the effect of NAO depends on latitude and, ultimately, SST. The strength of these findings is that they are derived from a comparative analysis of several populations of a dozen species of seabirds, rather than single colonies.

## Appendix A

**TableA1:** Studies of seabird breeding success used in the analyses

Species	English name	Colony	Cluster	Latitude	Longitude	Years	Length	Source
*Alca torda*	Razorbill	Isle of May	1	56.2°N	2.6°W	1982–1987	6	Harris & Wanless (1989)
*Calonectris diomedea*	Cory's shearwater	Selvagem Grande	2	30.2°N	15.9°W	1983–1996	14	Mougin *et al.* (2000)
*Fratercula arctica*	Atlantic puffin	Isle of May	3	56.2°N	2.6°W	1977–1990	14	Harris & Bailey (1992)
*Fratercula arctica*	Atlantic puffin	Dùn	4	57.8°N	8.6°W	1973–1994	17	Harris *et al.* (1998)
*Fratercula arctica*	Atlantic puffin	Runde	5	62.4°N	5.6°E	1980–1983	4	Barrett *et al.* (1987)
*Fulmarus glacialis*	Northern fulmar	Eynhallow	6	59.1°N	3.1°W	1958–1978	21	Ollason & Dunnet (1980)
*Larus argentatus*	Herring gull	Tryskärsgrund	7	60.0°N	23.0°E	1983–1988	6	Kilpi (1989)
*Larus fuscus*	Lesser black-backed gull	Söderskär	8	60.0°N	25.0°E	1980–1989	10	Hario (1990)
*Morus bassanus*	Northern gannet	Bempton	9	54.1°N	0.1°W	1961–1976	16	Nelson (1978)
*Morus bassanus*	Northern gannet	Bass Needle	10	56.2°N	2.6°W	1971–1976	6	Nelson (1978)
*Morus bassanus*	Northern gannet	Bass Rock 5	10	56.2°N	2.6°W	1970–1976	7	Nelson (1978)
*Morus bassanus*	Northern gannet	Bass Rock 6	10	56.2°N	2.6°W	1970–1973	4	Nelson (1978)
*Morus bassanus*	Northern gannet	Troup Head	11	57.7°N	2.3°W	1988–1995	8	Wanless *et al.* (1996)
*Pterodroma madeira*	Zino's petrel	Madeira	12	32.8°N	16.9°W	1986–2000	15	Zino *et al.* (2001)
*Puffinus puffinus*	Manx shearwater	Skokholm 1	13	51.7°N	5.3°W	1973–1976	4	Brooke (1978)
*Puffinus puffinus*	Manx shearwater	Skokholm 2	13	51.7°N	5.3°W	1973–1976	4	Brooke (1978)
*Puffinus puffinus*	Manx shearwater	Canna	14	57.0°N	6.6°W	1976–1997	22	Swann (1995); Swann (2000)
*Rissa tridactyla*	Black-legged kittiwake	S Shields	15	55.0°N	1.4°W	1954–1982	29	Coulson & Thomas (1985a, 1985b)
*Rissa tridactyla*	Black-legged kittiwake	Isle of May	16	56.2°N	2.6°W	1989–1996	8	Harris & Wanless (1997)
*Sterna hirundo*	Common tern	Little Island	17	39.6°N	74.2°W	1976–1980	5	Burger & Gochfeld (1991)
*Sterna hirundo*	Common tern	W Ham	17	39.6°N	74.2°W	1979–1985	6	Burger & Gochfeld (1991)
*Sterna hirundo*	Common tern	E Carvel	17	39.7°N	74.2°W	1976–1983	8	Burger & Gochfeld (1991)
*Sterna hirundo*	Common tern	Pettit	17	39.7°N	74.2°W	1980–1985	6	Burger & Gochfeld (1991)
*Sterna hirundo*	Common tern	W Carvel	17	39.7°N	74.2°W	1976–1980	5	Burger & Gochfeld (1991)
*Sterna hirundo*	Common tern	W Vol	17	39.7°N	74.1°W	1976–1985	9	Burger & Gochfeld (1991)
*Sterna hirundo*	Common tern	N Lavallette	17	39.8°N	74.1°W	1976–1984	9	Burger & Gochfeld (1991)
*Sterna hirundo*	Common tern	NW Lavallette	17	39.8°N	74.1°W	1976–1979	4	Burger Gochfeld (1991)
*Sterna hirundo*	Common tern	S Lavallette	17	39.8°N	74.1°W	1976–1979	4	Burger & Gochfeld (1991)
*Sterna hirundo*	Common tern	SW Lavallette	17	39.8°N	74.1°W	1976–1984	9	Burger & Gochfeld (1991)
*Sterna hirundo*	Common tern	Minsener Oldeoog	18	53.8°N	8.0°E	1981–1996	16	Becker (1998)
*Sterna paradisaea*	Arctic tern	Machias Seal Island	19	44.5°N	67.1°W	1978–1981	4	Newell (1985)
*Uria aalge*	Common murre	Isle of May	20	56.2°N	2.6°W	1981–1998	18	Harris & Bailey (1992); Rindorf *et al.* (2000)
*Uria aalge*	Common murre	Stora Karlsö	21	57.3°N	18.0°E	1974–1977	4	Hedgren (1980)

‘Cluster’ refers to geographically close colonies. Time series with the same ‘cluster’ number were combined in certain analyses (see ‘Material and methods’, and test 7 of Table A2).

**TableA2:** Variations of the models presented in Table 2

Model	*F*	*P*	df	*r*^2^	ΔAIC_C_
(1) Weighting each slope by the inverse variance of the underlying time series					
Latitude	9.17	0.0049	1, 31	0.23	−6.12
Latitude × taxon	3.77	0.0048	9, 23	0.60	+0.26
Local correlation with SST	17.70	0.00020	1, 31	0.36	−12.48
(2) Excluding slopes derived from times series shorter than 5 years					
Latitude	14.50	0.00091	1, 23	0.39	–9.62
Latitude × taxon	2.22	0.082	9, 15	0.57	+16.57
Local correlation with SST	19.53	0.00020	1, 23	0.46	–12.77
(3) Excluding slopes derived from times series shorter than 6 years					
Latitude	12.69	0.0018	1, 21	0.38	–8.21
Latitude × taxon	1.90	0.14	9, 13	0.57	+22.07
Local correlation with SST	17.31	0.00044	1, 21	0.45	–11.16
(4) Using NAO slopes derived from multivariate analysis including both NAO and SST*					
Latitude	9.69	0.0040	1, 31	0.24	–6.55
Latitude × taxon	2.01	0.085	9, 23	0.44	+11.02
Local correlation with SST	14.53	0.00062	1, 31	0.32	–10.26
(5) Using slopes derived from first year differentials of the time series rather than raw data*					
Latitude	4.68	0.041	1, 23	0.17	–2.03
Latitude × taxon	3.26	0.021	9, 15	0.66	+10.68
Local correlation with SST	18.29	0.00028	1, 23	0.44	–12.03
(6) Using robust fitting of linear models (R function rlm; Venables & Ripley, 2002)					
Latitude	10.80	0.0025	1, 31	0.26	–7.41
Latitude × taxon	3.47	0.0076	9, 23	0.58	+2.00
Local correlation with SST	15.10	0.00050	1, 31	0.33	–10.64
(7) Treating geographically close colonies as single data points					
Latitude	3.57	0.074	1, 19	0.16	–0.87
Latitude × taxon	1.79	0.18	9, 11	0.59	+27.70
Local correlation with SST	5.57	0.029	1, 19	0.23	–2.66

The three models from Table 2 were rerun using different methods or under different statistical assumptions. Results are largely in agreement with Table 2 and each other. Only model statistics are provided. See Table 1 for further explanations

* The slopes were *z*-transformed (Sokal & Rohlf, 1995, p. 575) prior to analyses in all but two cases: in analysis 4, *z*-transformation was neither applicable nor necessary (the distribution did not differ from normality and slopes were processed untransformed); in analysis 5, *z*^*^-transformation (Sokal & Rohlf, 1995, p. 579) was required to yield approximately normally distributed values.
